# Supplementary material for: Effects of Different Lengths of Oligo (Ethylene Glycol) Side Chains on the Electrochromic and Photovoltaic Properties of Benzothiadiazole-Based Donor-Acceptor Conjugated Polymers
Source: Molecules. 2023 Feb 22;28(5):2056. doi: 10.3390/molecules28052056 (PMC10004708; doi:10.3390/molecules28052056)
Supplement: Supplementary file 1 [file molecules-28-02056-s001.zip › molecules-2174750-supplementary.pdf]

Supplementary Materials

Effects of Different Lengths of Oligo (Ethylene Glycol) Side Chains on the Electrochromic and Photovoltaic Properties of Benzothiadiazole-Based Donor-Acceptor Conjugated Polymers

Songrui Jia[1], Shiying Qi[1,2], Zhen Xing[1], Shiyi Li[1], Qilin Wang[1] and Zheng Chen[1,*]

[1] Key of High-Performance Plastics, Ministry of Education, National & Local Joint Engineering Laboratory for Synthesis Technology of High-Performance Polymer, College of Chemistry, Jilin University, Changchun 130012, China

[2] Department of Materials Science, Fudan University, Shanghai 200433, People's Republic of China

[*] Correspondence: chenzheng2013@jlu.edu.cn

Table of contents

1. Synthesis of compounds 3-8

2. ^{1}H NMR spectra of monomers and PBDT-DTBF polymers 8-14

3. Mass spectrum of monomer 12 14

4. FTIR spectra of PBDT-DTBF polymers 15

5. TGA curves of PBDT-DTBF polymers 15

6. Cyclic voltammogram reference 16

7. The Mott-Schottky plots 16

8. The Nyquist plots 16

9. Spectroelectrochemical tests 17

10. Color coordinate changes of PBDT-DTBF films 17

11. Cyclical stability and switching time of PBDT-DTBF films 18

1. Synthesis

1.1 Synthesis of Compound 1 [1].

In the ice water bath, diethylene glycol monomethyl ether (12.01 g, 0.10 mol) was added to the 500 mL three-neck flask, 150 mL of tetrahydrofuran (THF) and 30 mL of deionized water were added to the reaction system and then sodium hydroxide (NaOH) (6.00 g, 0.15 mol) was added to the reaction system. After 0.5 hours, the toluene sulfonyl chloride (20.97 g, 0.11 mol) was dissolved in 60 mL THF and slowly added to the reaction system with a constant pressure drop funnel. After 4 hours, the crude product was quenched in saturated sodium chloride (NaCl) solution and repeatedly extracted with dichloromethane (CH_2Cl_2) and anhydrous magnesium sulfate ($MgSO_4$). After the organic solvent was removed by rotating evaporator and the crude product was purified by silica gel column chromatography using ethyl acetate / petroleum ether (1:1), 15.18 g of light-yellow liquid was obtained, yield: 55%. ^{1}H NMR (300 MHz, Chloroform-*d*) δ = 7.79 (d, *J*=8.3, 2H), 7.34 (d, *J*=7.2, 2H), 4.16 (m, 2H), 3.68 (m, 2H), 3.57 (m, 2H), 3.48 (m, 2H), 3.34 (s, 3H), 2.44 (s, 3H).

1.2 Synthesis of Compound 2 [2].

Triethylene glycol mono-methyl ether (16.42 g, 0.10 mol) was dissolved in 150 mL THF, and then NaOH (6.00 g, 0.15 mol) was slowly added to maintain the reaction at 0 °C under an ice-water bath. Then, 30 mL water was added to the reaction. After the solution was stirred for 5 minutes, the para-toluene sulfonyl chloride (20.97 g, 0.11 mol) was dissolved in 30 mL THF and slowly added to the reaction system with a constant pressure drop funnel. After 1 hour, the ice-cold water bath was removed and the reaction was reacted at room temperature for 5 hours, then poured into 100 mL of saturated NaCl solution, and the products were extracted with CH_2Cl_2 and dried with anhydrous $MgSO_4$. The crude product was purified by silica gel column chromatography using ethyl acetate / petroleum ether (1:1). Finally, 18.14 g of yellowish oily product was obtained with a yield: 57%. ^{1}H NMR (300 MHz, Chloroform-d) δ = 7.78 (d, J=8.3 Hz, 2H), 7.32 (d, J=9.3 Hz, 2H), 4.16 (m, 2H), 3.67 (m, 2H), 3.59 (m, 6H), 3.52 (m, 2H), 3.35 (s, 3H), 2.44 (s, 3H).

1.3 Synthesis of Compound 3 [3].

Benzo [1,2-b: 4,5-b '] dithiophene-4,8-diketone (2.20 g, 0.01 mol), zinc powder (2.29 g, 0.04 mol), NaOH (6.00 g, 0.15 mol) and 100 mL of water were added to a 500 mL three-neck flask and heated under argon protection to 100 °C and the solution was colored yellow-green. After heating for 4 hours, the reaction was cooled down to 50 °C before adding the phase transfer catalyst tetra-butyllammonium bromide (TBAB) (0.19 g, 0.60 mmol) to the reaction system, followed by methyl para-toluene sulfonate (7.45 g, 0.04 mol) and heated overnight. The mixture was extracted three times with trichloromethane ($CHCl_3$), the organic phase was merged, dried with an anhydrous $MgSO_4$, and the solvent was removed with a rotary evaporator. The crude product was purified by silica gel column chromatography using dichloromethane / petroleum ether (1:1) to obtain 1.76 g of white solid with a yield: 70%. ^{1}H NMR (300 MHz, Chloroform-d) δ = 7.51 (d, J=5.6, 1H), 7.40 (d, J=5.6, 1H), 4.14 (s, 3H).

1.4 Synthesis of Compound 4 [4].

Benzo [1,2-b: 4,5-b '] dithiophene-4,8-diketone (2.20 g, 10.00 mmol), zinc powder (2.29 g, 35.00 mmol), NaOH (6.00 g, 150.00 mmol) and 100 mL of water were added to a 500 mL three-neck flask and heated under argon protection up to 100 °C and the solution was colored yellow-green. After heating for 4 hours, TBAB (0.19 g, 0.60 mmol) was added backward to the reaction system, followed by compound 1 (8.78 g, 32.00 mol) and heated overnight. The mixture was extracted three times with $CHCl_3$, the organic phase was merged, dried and anhydrous, and the solvent was removed with a rotary evaporator. The crude product was purified by silica gel column chromatography using ethyl acetate / petroleum ether (1:1) to obtain 2.40 g of a pink solid with a yield of 56%. ^{1}H NMR (300 MHz, Chloroform-d) δ = 7.56 (d, J=5.5, 2H), 7.35 (d, J=5.5, 2H), 4.44 (m, 4H), 3.87 (m, 4H), 3.74 (m, 4H), 3.60 (m, 4H), 3.40 (s, 6H).

1.5 Synthesis of Compound 5 [4].

Benzo [1,2-b: 4,5-b '] dithiophene-4,8-dione (2.20 g, 10.00 mmol), zinc powder (2.29 g, 35.00 mmol), sodium hydroxide (6.00 g, 150.00 mmol) and 100 mL water were

added to a 500 mL three-neck flask, heated under argon protection to 100 °C and the solution colored yellow-green. After heating for 4 hours, TBAB (0.19 g, 0.60 mmol) was added backward to the reaction system, followed by compound 2 (11.14 g, 35.00 mol) and heated overnight. The mixture was extracted three times with $CHCl_3$, the organic phase was merged, dried with an anhydrous $MgSO_4$, and the solvent was removed with a rotary evaporator. The crude product was purified by silica gel column chromatography using ethyl acetate / petroleum ether (4:1) to obtain 2.50 g of a pink solid with a yield of 51%. ^{1}H NMR (300 MHz, Chloroform-d) δ = 7.55 (d, J=5.6, 2H), 7.35 (d, J=5.5, 2H), 4.41 (m, 4H), 3.85 (m, 4H), 3.71 (m, 12H), 3.54 (m, 4H), 3.36 (s, 6H).

1.6 Synthesis of Monomer 6 [5].

Compound 3 (2.50 g, 10.00 mmol) and 25 mL of anhydrous THF were loaded into an argon-filled 100 mL reaction flask; the liquid nitrogen acetone bath cooled the reaction to –78 °C for a period, 8.8 mL of n-butyllithium (22 mmol, 2.5 mol/L in Hexanes) was injected into the system through a syringe for 40 minutes and the reaction was allowed to continue at room temperature for one hour. After that, the liquid nitrogen acetone bath reaction continued to cool the reaction to –78 °C for 15 minutes, and 23 mL of trimethyltin chloride (23.00 mmol, 1.0 mol/L in Hexanes) was injected with a syringe for 30 minutes. The reaction was conducted at room temperature and the solution turned yellow. After the overnight reaction at room temperature, the solution became brown, and after being injected with saturated potassium fluoride (KF) solution was quenched, the organic phase was extracted with $CHCl_3$, the organic phase was dried with anhydrous Na_2SO_4, the organic solvent was removed by the rotary evaporator, the crude product was frozen and recrystallized with methanol, and finally 2.82 g of white needle solid was obtained, yield: 49%. ^{1}H NMR (300 MHz, Chloroform-d) δ = 7.54 (s, 2H), 4.15 (s, 6H), 0.45 (s, 18H).

1.7 Synthesis of Monomer 7 [4].

Compound 4 (4.27 g, 10.00 mmol) and 25 mL of anhydrous THF were loaded into

a 100 mL reaction flask filled with argon, and then liquid nitrogen acetone bath cooled the reaction to –78 ° C. After maintaining the reaction system at –78 °C for a certain period of time, 8.8 mL n-butyl lithium (22.00 mmol, 2.5 mol/L in Hexanes) was injected by syringe into the reaction system. After 40 min of the reaction, the liquid nitrogen acetone bath was removed to allow the reaction to recover to room temperature and to react for 1 h at room temperature. After that, the liquid nitrogen acetone bath reaction was used to cool the reaction to-78 °C for 15 minutes, 23 mL of trimethyltin chloride (23.00 mmol, 1.0mol/L in Hexanes) was injected with a syringe and after maintaining -78 °C for 30 minutes, the reaction was performed at room temperature and the solution turned yellow. After that, the reaction was placed at room temperature overnight and the solution became brown. The saturated KF solution was injected and quenched, and the organic phase was extracted with CH_2Cl_2. After the organic phase was dried with anhydrous sodium sulfate, the organic solvent was removed by the rotary evaporator, and the crude product was recrystallized with methanol. Finally, 1.96 g of white needle solid was obtained with a yield of 26%. ^{1}H NMR (300 MHz, Chloroform-d) δ = 7.58 (s, 2H), 4.47 (t, J=4.8, 4H), 3.89 (m, 4H), 3.76 (m, 4H), 3.63 (m, 4H), 3.41 (s, 6H), 0.44 (s, 18H).

1.8 Synthesis of Monomer 8 [4].

Compound 5 (5.15 g, 10.00 mmol) and 25 mL of anhydrous THF were loaded into a 100 mL reaction bottle filled with argon, which was cooled to –78 °C for a while. Then, 8.8 mL of n-butyllithium (22.00 mmol, 2.5 mol/L in Hexanes) was removed to cool the reaction to room temperature for one hour. After that, the liquid nitrogen acetone bath reaction was continued to cool the reaction to –78 °C for 15 minutes, 23 mL of trimethyltin chloride (23.00 mmol, 1.0 mol/L in Hexanes) was injected with a syringe for 30 minutes, and the room temperature reaction and the solution turned yellow. After the overnight reaction at room temperature, the solution became brown, quenched with potassium fluoride solution. Then the organic phase was extracted with CH_2Cl_2, the organic phase was dried with anhydrous Na_2SO_4, the organic solvent was

removed by the rotary evaporator, the crude product was frozen and recrystallized with ethanol and it finally received a white needle: 0.76 g solid, a yield of 9%. ^{1}H NMR (300 MHz, Chloroform-d) δ = 7.56 (s, 2H), 4.45 (m, 4H), 3.90 (d, J=5.0, 4H), 3.78 (m, 4H), 3.73 (m, 4H), 3.67 (m, 4H), 3.55 (m, 4H), 3.37 (s, 6H), 0.44 (s, 18H).

1.9 Synthesis of Compound 9 [2].

The 3-thiophene ethanol (12.82 g, 0.10 mol) was dissolved in 150 mL DMF, tert-butanol potassium (12.34 g, 0.11 mol) was slowly added, and the reaction temperature was maintained at –5 °C with an ice-cold salt bath. After stirring the solution for 30 min, a solution was slowly added of compound 2 (19.10 g, 0.06 mol) in 30 mL DMF. After 1 h, the ice-cold salt bath was removed and the mixture was stirred overnight, before being poured and quenched in a saturated $NaHCO_3$ solution. Products were extracted with CH_2Cl_2 and dried with anhydrous Na_2SO_4. Finally, 5.29 g of yellow oily liquid was obtained at 230°C, with a yield of 32%. ^{1}H NMR (300 MHz, Chloroform-d) δ = 7.24 (dd, J=4.7, 3.2, 1H), 7.03 (dd, J=3.0, 0.7, 1H), 6.97 (dd, J=4.9, 1.7, 1H), 3.64 (m, 12H), 3.55 (m, 2H), 3.38 (d, J=0.5, 3H), 2.92 (t, J=7.0, 2H).

1.10 Synthesis of Compound 10.

In an anaerobic, argon atmosphere, compound 9 (5.48 g, 0.02 mol) was dissolved in 40 mL anhydrous THF, n-butyl lithium (1.35 g, 0.02 mol) was slowly added, and the liquid nitrogen acetone bath was kept below –78 °C. After 40 min, the mixture was allowed to stir for 20 min at room temperature. Then, tributyl tin chloride (7.48 g, 0.02 mol) was added to the mixture, and the temperature was kept at –78 ° C. After 30 minutes, the mixture was stirred overnight, and then poured into 10 mL of saturated potassium fluoride (KF) solution. They were then extracted three times with CH_2Cl_2. Finally, the organic layer was removed by anhydrous Na_2SO_4. The final product was 8.92 g of brown mucus. ^{1}H NMR (300 MHz, $CDCl_3$): δ=7.53 (d, J = 4.7 Hz, 1H), 7.14 (d, J = 4.7 Hz, 1H), 3.67 (s, 12H), 3.55 (s, 2H), 3.38 (s, 3H), 2.91 (s, 2H), 1.54 (s, 6H), 1.34 (s, 6H), 1.11 (s, 6H), 0.89 (s, 9H).

1.11 Synthesis of Compound 11 [6].

The 4,7-dibromine-5,6-difluoride-2,1,3-benzothiadiazole (1.00 g, 3.03 mmol) and

three (dibenzyl acetone) dipalladium chloroform adduct (Pb_2 $(dba)_3 \cdot CHCl_3$) (0.25 g, 0.24 mmol), Tris(*o*-tolyl) phosphine (P (*o*-tol)$_3$) (0.59 g, 1.94 mmol) were added to two flasks containing 25 mL anhydrous toluene. Immediately, tributyl(4-(2-(2-(2-methoxyethoxy)ethoxy)ethoxy)thiophen-2-yl)stannane (5.12 g, 9.09 mmol) was added to two-neck flasks. The solution was heated to 110 °C and stirred continuously for 12 hours. Then, the mixture was quenched into a saturated potassium fluoride (KF) solution and extracted three times with CH_2Cl_2. The crude product was purified by silica gel column chromatography, and the eluent was MeOH / EA=1:50. Finally, the orange-yellow oily liquid was obtained, 0.74 g, yield: 34%. ^{1}H NMR (300 MHz, $CDCl_3$): δ = 7.57 (d. J = 5.2 Hz, 2H), 7.22 (d, J = 5.2 Hz, 2H), 3.67-3.53 (m, 28H), 3.37(s, 1H), 2.87-2.82 (t, J = 7.2 Hz, 4H).

1.12 Synthesis of Monomer 12 [7].

The compound 11 (1.00 g, 1.39 mmol) and N-bromoosuccinimide (NBS) (0.54 g, 3.06 mmol) were added to a 25 mL flask containing 10 mL $CHCl_3$ and 5 mL glacial acetic acid (AcOH). The solution was heated to 60 °C and stirred continuously for 12 hours. Then, the mixture was quenched into a saturated potassium fluoride (KF) solution and extracted three times with CH_2Cl_2. Water was removed from the organic solvents with anhydrous $MgSO_4$. The crude product was purified by silica gel column chromatography with the eluent MeOH / EA=1:50, and, finally, 0.24 g orange oil liquid was produced with yield: 20%. ^{1}H NMR (300 MHz, Chloroform-d): δ = 7.22(s, 2H), 3.67-3.51 (m, 28H), 3.36 (s, 4H), 2.79-2.75 (t, J=6.8 Hz, 4H); HRMS: Found: $[M+H]^+$ 873.0313; Molecular formula: $C_{32}H_{41}Br_2F_2N_2O_8S_3$; Requires: $[M+H]^+$ = 873.0354.

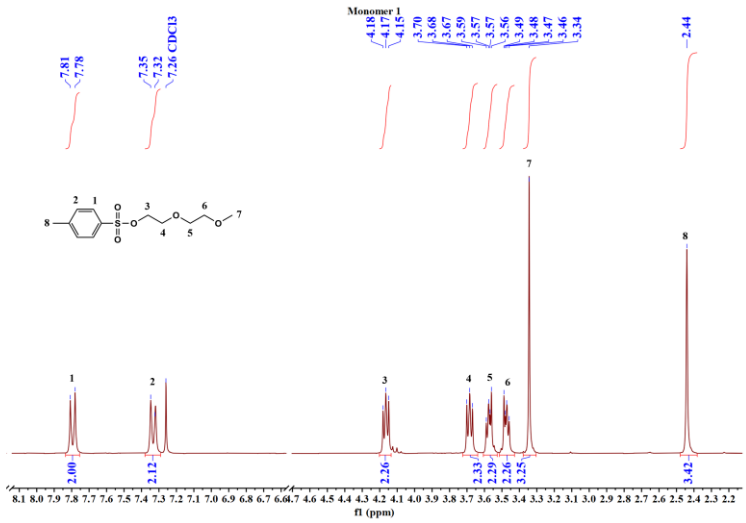

Figure S1. ^{1}H NMR spectra of compound 1.

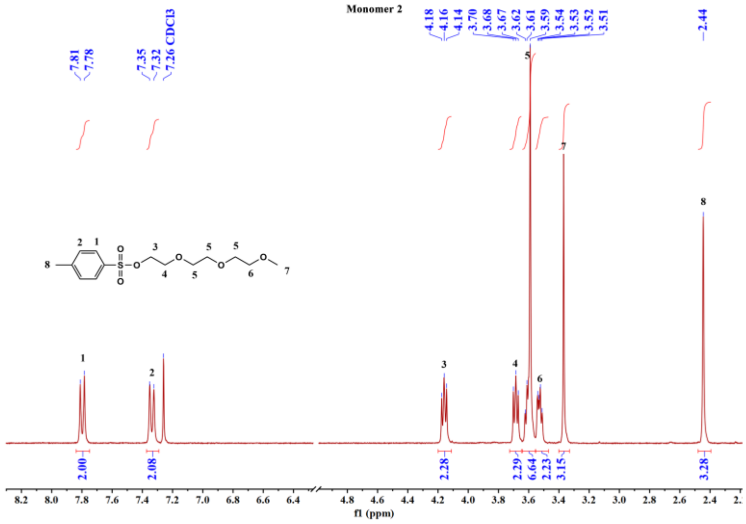

Figure S2. ^{1}H NMR spectra of compound 2.

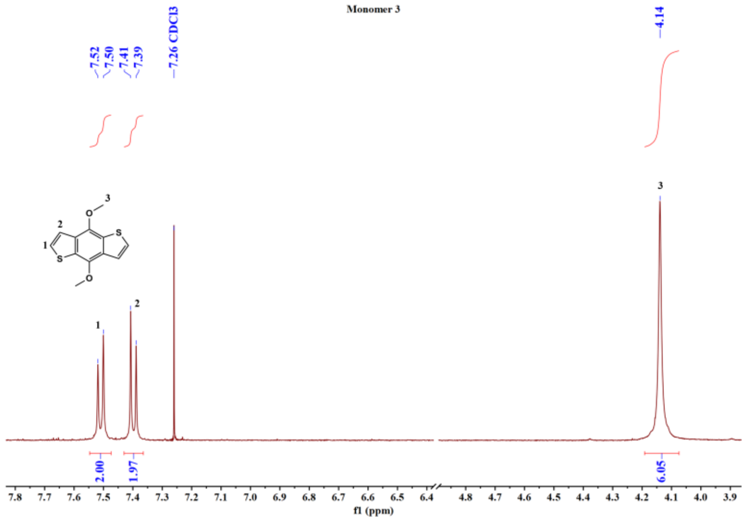

Figure S3. ^{1}H NMR spectra of compound 3.

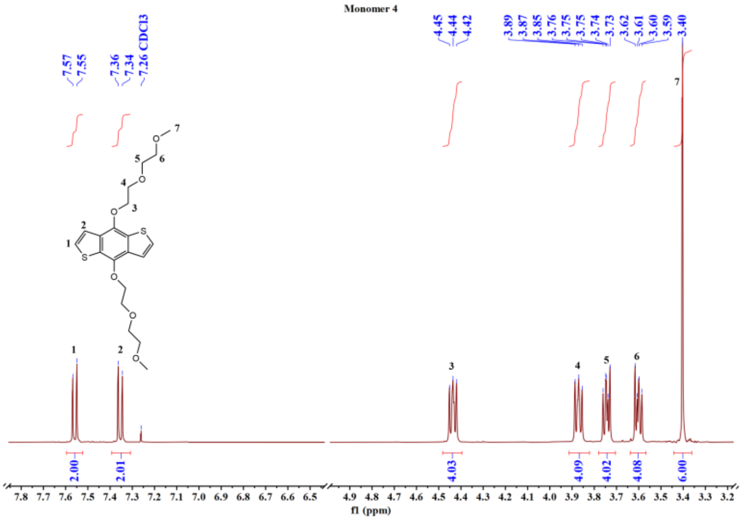

Figure S4. ^{1}H NMR spectra of compound 4.

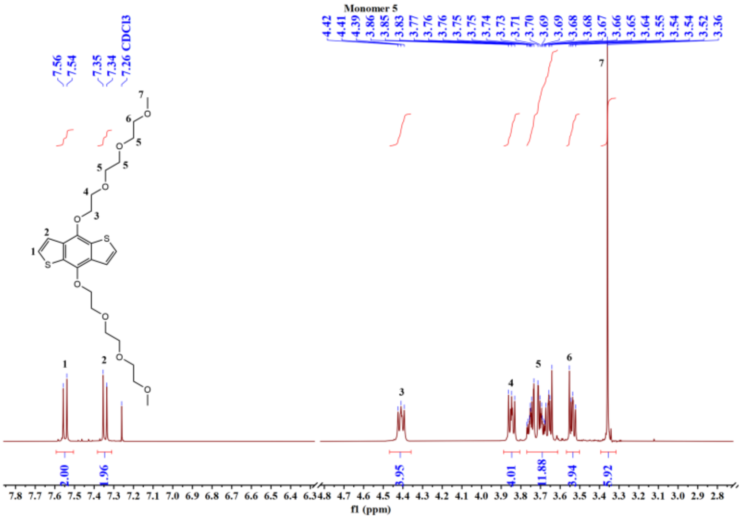

Figure S5. ^{1}H NMR spectra of compound 5.

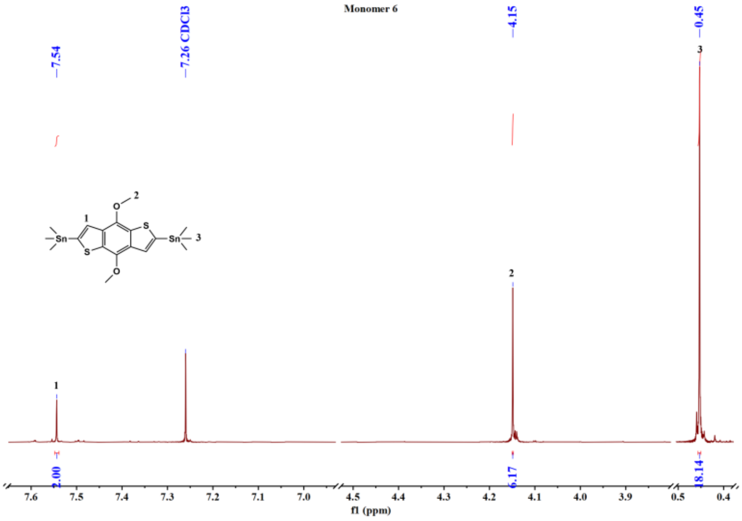

Figure S6. ^{1}H NMR spectra of monomer 6.

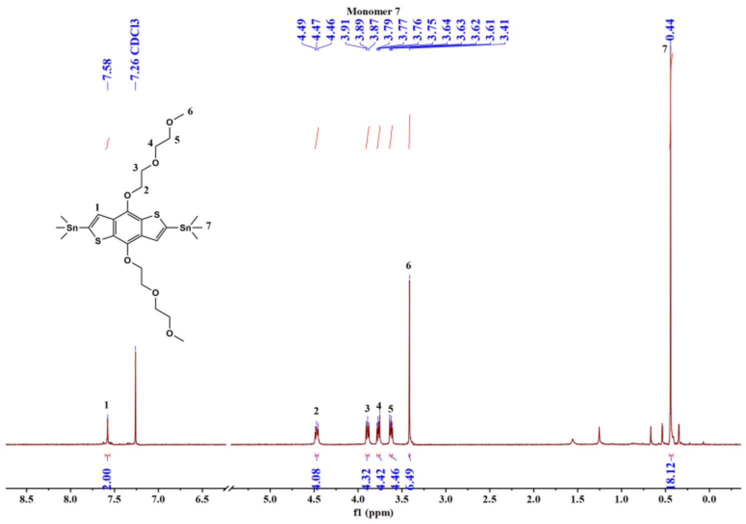

Figure S7. ^{1}H NMR spectra of monomer 7.

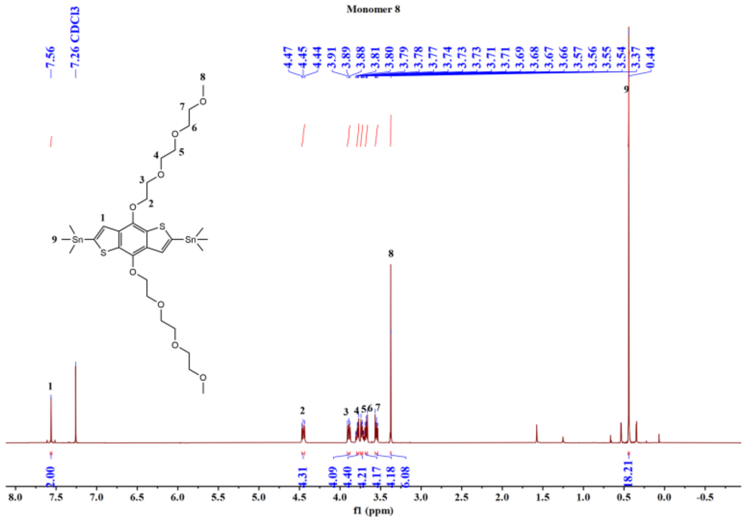

Figure S8. ^{1}H NMR spectra of monomer 8.

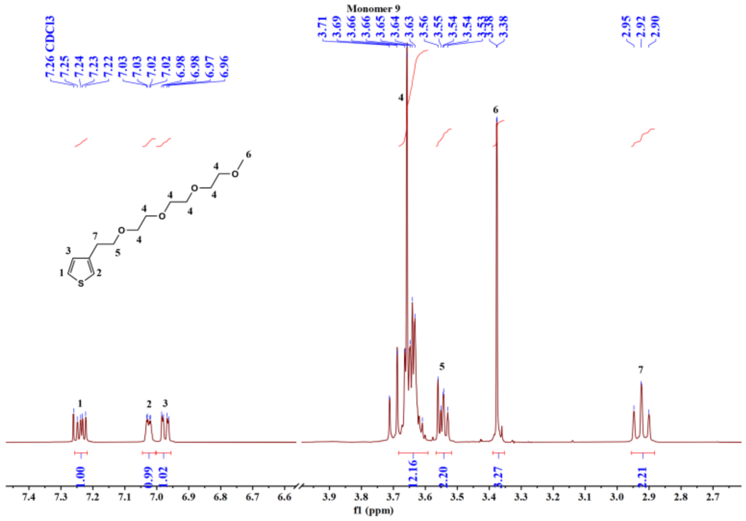

Figure S9. ^{1}H NMR spectra of compound 9.

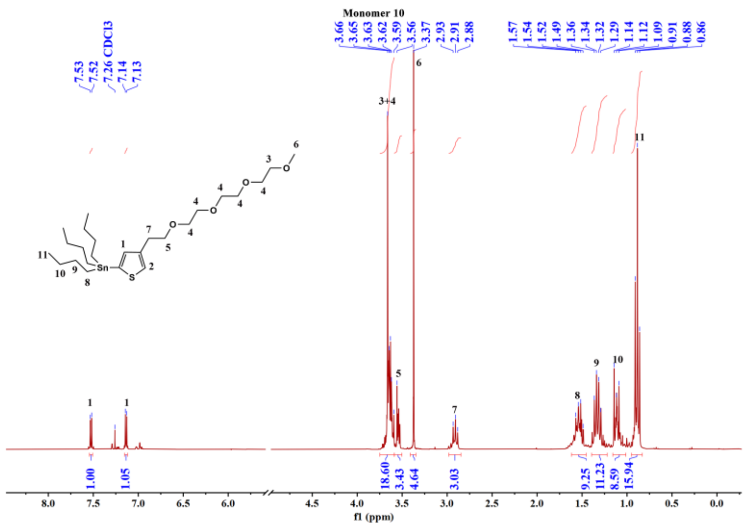

Figure S10. ^{1}H NMR spectra of compound 10.

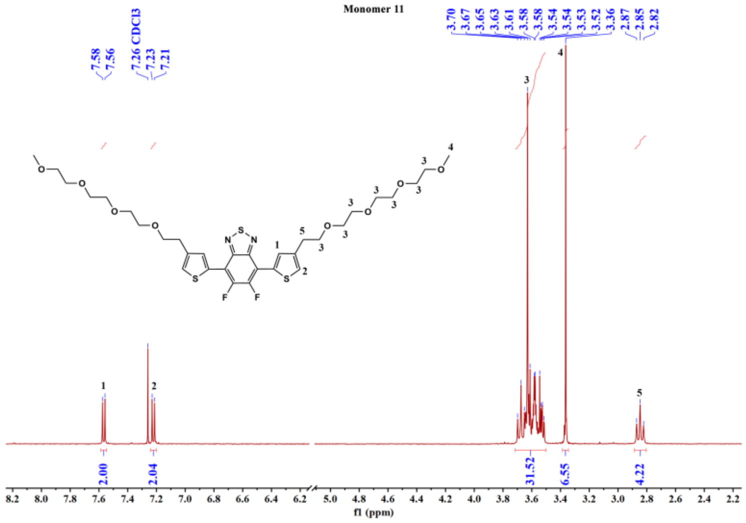

Figure S11. ^{1}H NMR spectra of compound 11.

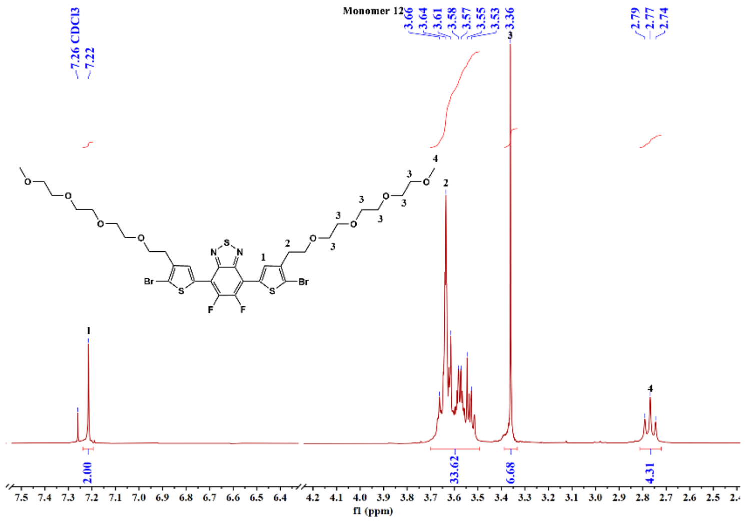

Figure S12. ^{1}H NMR spectra of monomer 12.

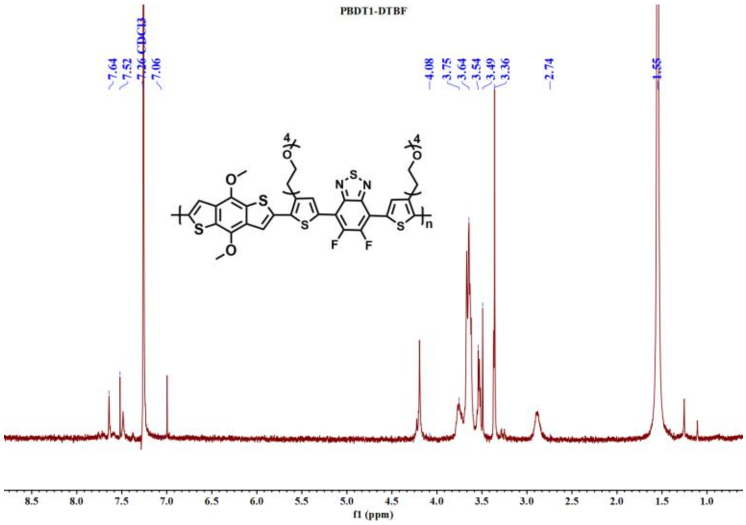

Figure S13. ^{1}H NMR spectra of polymer PBDT1-DTBF.

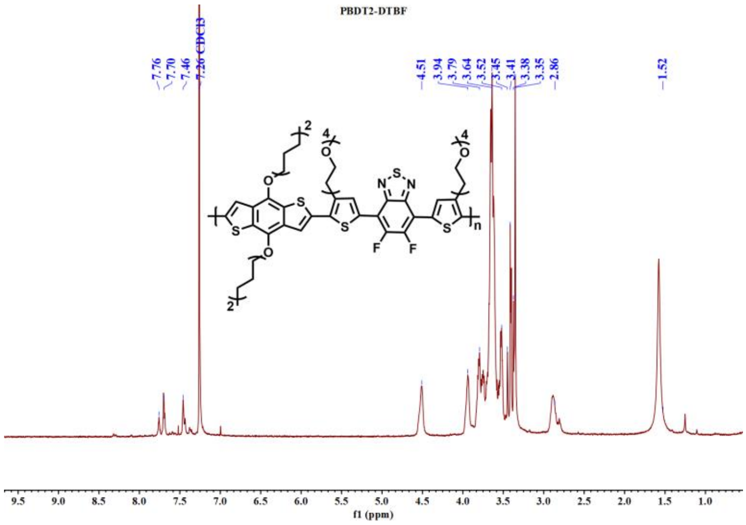

Figure S14. ^{1}H NMR spectra of polymer PBDT2-DTBF.

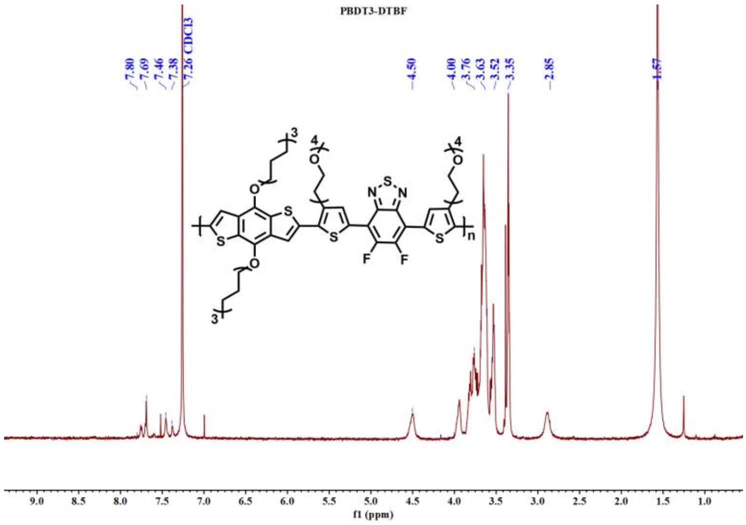

Figure S15. ^{1}H NMR spectra of polymer PBDT3-DTBF.

Acquisition Parameter

Source Type	ESI	Ion Polarity	Positive	Set Nebulizer	0.6 Bar
Focus	Active	Set Capillary	4500 V	Set Dry Heater	180 °C
Scan Begin	50 m/z	Set End Plate Offset	-500 V	Set Dry Gas	8.0 l/min
Scan End	3000 m/z	Set Collision Cell RF	150.0 Vpp	Set Divert Valve	Waste

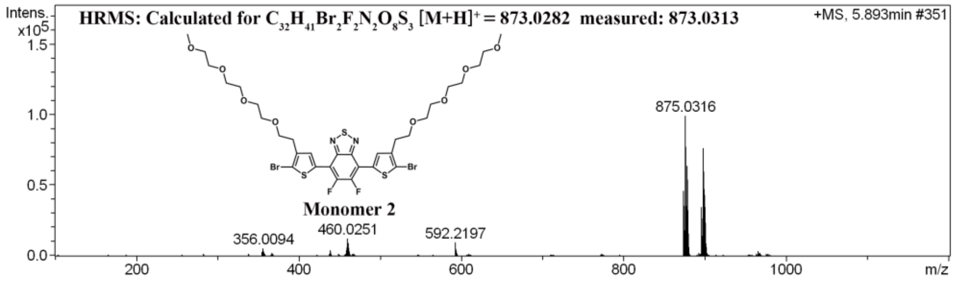

Meas. m/z	#	Formula	Score	m/z	err [mDa]	err [ppm]	mSigma	rdb	e⁻ Conf	N-Rule
873.0313	1	C 32 H 41 Br 2 F 2 N 2 O 8 S 3	18.66	873.0354	4.1	4.8	9.5	11.5	even	ok
	2	C 28 H 41 Br 2 F 2 N 2 O 11 S 3	0.00	873.0202	-11.1	-12.7	10.1	7.5	even	ok
	3	C 35 H 37 Br 2 F 2 N 2 O 6 S 3	0.00	873.0143	-17.0	-19.5	21.3	16.5	even	ok
	4	C 36 H 41 Br 2 F 2 N 2 O 5 S 3	0.00	873.0507	19.4	22.2	25.6	15.5	even	ok
	5	C 39 H 37 Br 2 F 2 N 2 O 3 S 3	100.00	873.0296	-1.7	-2.0	38.0	20.5	even	ok
	6	C 43 H 37 Br 2 F 2 N 2 S 3	0.00	873.0448	13.5	15.5	54.8	24.5	even	ok

Figure S16. Mass spectrum of monomer 12.

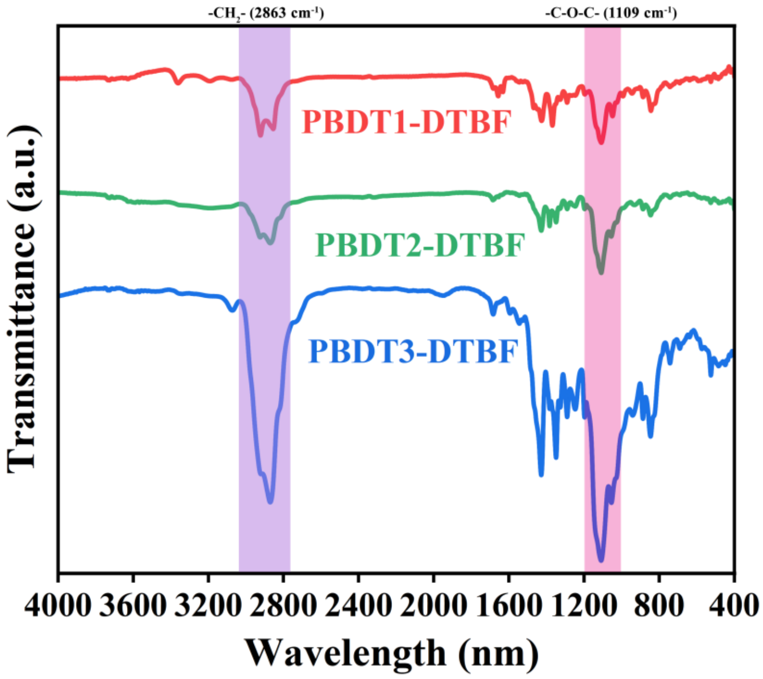

Figure S17. FTIR spectra of the polymers PBDT1-DTBF, PBDT2-DTBF and PBDT3-DTBF.

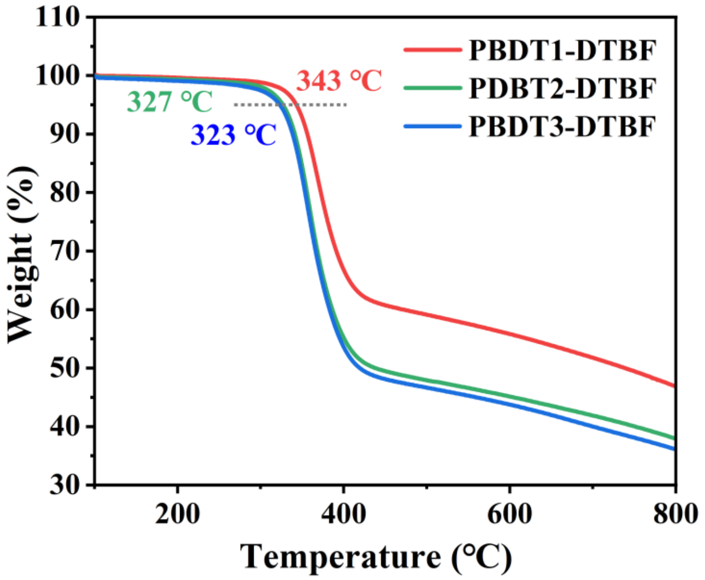

Figure S18. TGA curves of polymers PBDT1-DTBF, PBDT2-DTBF and PBDT3-DTBF.

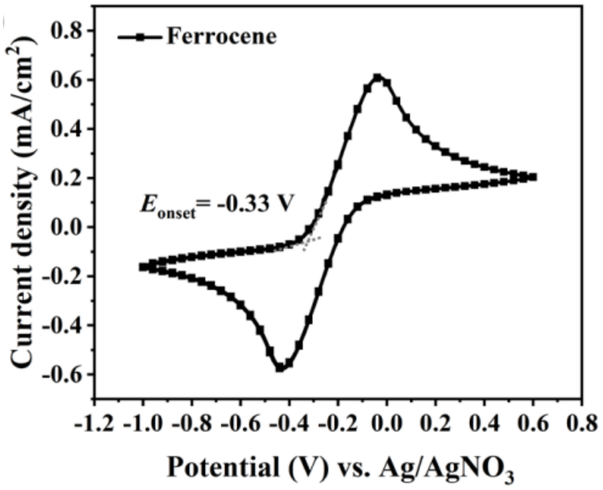

Figure S19. Cyclic voltammograms of Fc/Fc$^+$ reference

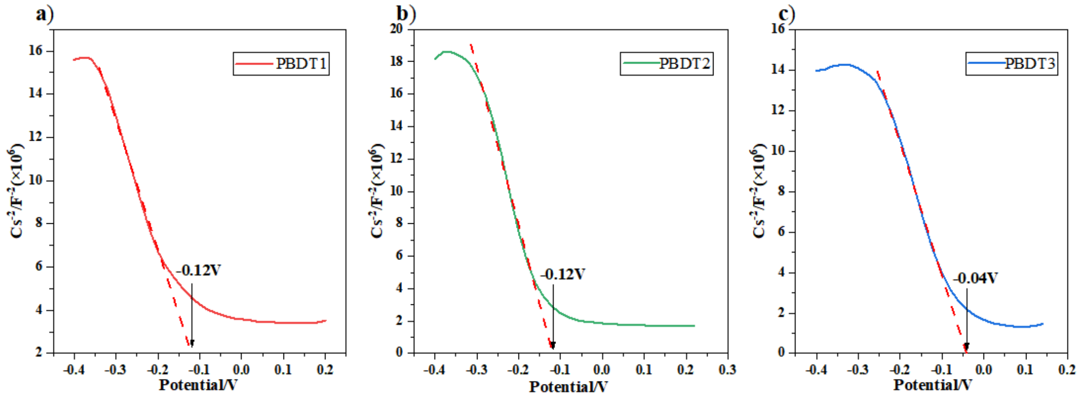

Figure S20. Mott–Schottky plots of (a) PBDT1-DTBF, (b) PBDT2-DTBF and (c) PBDT3-DTBF film processing from THF.

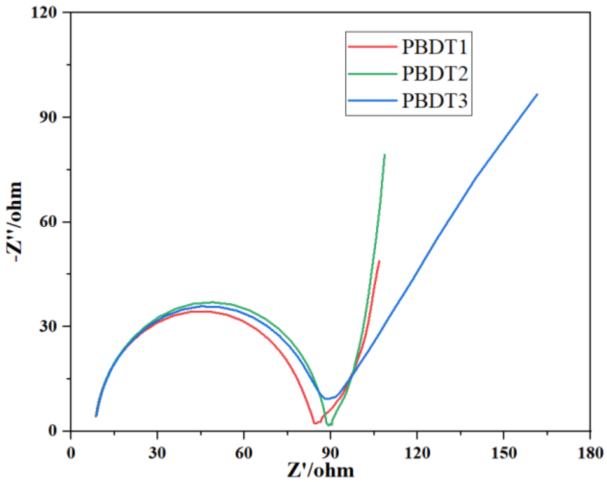

Figure S21. Nyquist plots of (a) PBDT1-DTBF, (b) PBDT2-DTBF and (c) PBDT3-DTBF film processing from THF.

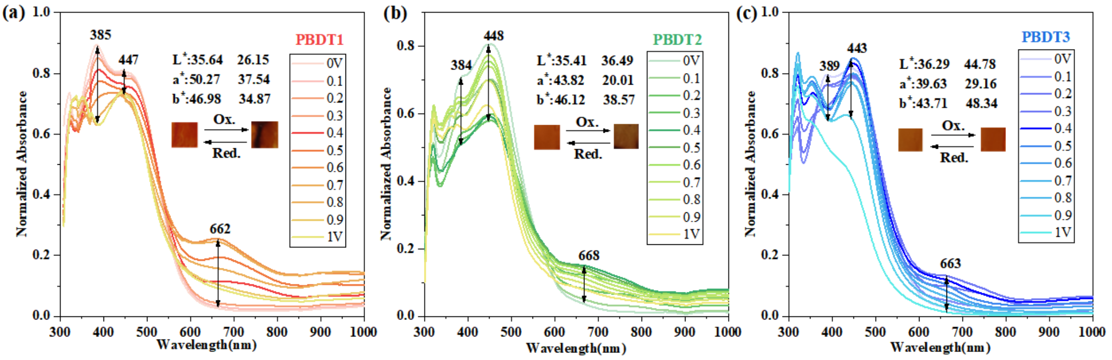

Figure S22. Spectroelectrochemical tests of (a) PBDT1-DTBF, (b) PBDT2-DTBF and (c) PBDT3-DTBF film processing from THF.

Table S1. Colorimetric analysis of the polymer films.

		L*, a*, b* color coordinates[a]		
polymer	solvent	neutral state	oxidized state	ΔE*[b]
PBDT1	CB	49.64, 16.31, 51.03	57.39, 13.26, 54.51	9.01
PBDT2		55.15, 1.68, 38.37	61.73, -3.21, 44.62	10.3
PBDT3		57.47, 1.32, 38.39	52.74, -1.92, 36.83	5.9
PBDT1	THF	35.64, 50.27, 46.98	26.15, 37.54, 34.87	19.9
PBDT2		35.41, 43.82, 46.12	36.49, 20.01, 38.57	25.0
PBDT3		36.29, 39.63, 43.71	44.78, 29.16, 48.34	14.3

[a] CIE 1976 L*a*b* color standards. [b] $\Delta E^* = \sqrt{(\Delta L^*)^2 + (\Delta a^*)^2 + (\Delta b^*)^2}$.

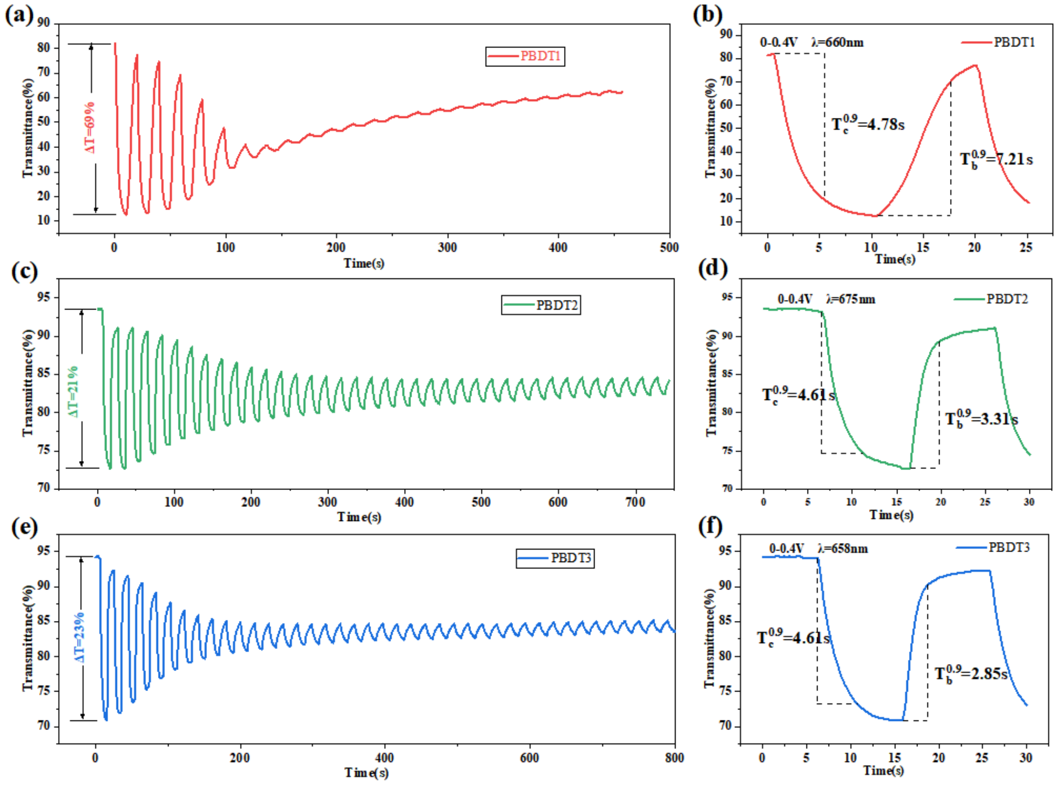

Figure S23. Cyclical stability and switching time of (a), (b) PBDT1-DTBF and (c), (d) PBDT2-DTBF and (e), (f) PBDT3-DTBF processing from THF.

References

1. Harjani, J.R.; Liang, C.; Jessop, P.G. A synthesis of acetamidines. *J. Org. Chem.* **2011**, *76*, 1683-1691, doi:10.1021/jo102358d.
2. Vallee, M.R.J.; Majkut, P.; Wilkening, I.; Weise, C.; Muller, G.; Hackenberger, C.P.R. Staudinger-Phosphonite reactions for the chemoselective transformation of azido-containing peptides and proteins. *Org. Lett.* **2011**, *13*, 5440-5443, doi:10.1021/ol2020175.
3. Wang, Y.F.; Parkin, S.R.; Watson, M.D. Benzodichalcogenophenes with perfluoroarene termini. *Org. Lett.* **2008**, *10*, 4421-4424, doi:10.1021/ol801569m.
4. Giovannitti, A.; Nielsen, C.B.; Rivnay, J.; Kirkus, M.; Harkin, D.J.; White, A.J.P.; Sirringhaus, H.; Malliaras, G.G.; McCulloch, I. Sodium and potassium ion selective conjugated polymers for optical ion detection in solution and solid state. *Adv. Funct. Mater.* **2016**, *26*, 514-523, doi:10.1002/adfm.201503791.
5. Kolanji, K.; Postulka, L.; Wolf, B.; Lang, M.; Schollmeyer, D.; Baumgarten, M. Planar Benzo[1,2-b:4,5-b ']dithiophene derivatives decorated with nitronyl and imino nitroxides. *J. Org. Chem.* **2019**, *84*, 140-149, doi:10.1021/acs.joc.8b02499.
6. Wang, N.; Chen, Z.; Wei, W.; Jiang, Z.H. Fluorinated Benzothiadiazole-Based Conjugated Polymers for high-performance polymer solar cells without any processing additives or post-treatments. *J. Am. Chem. Soc.* **2013**, *135*, 17060-17068, doi:10.1021/ja409881g.
7. Wang, C.; Li, C.; Wen, S.P.; Ma, P.F.; Wang, G.; Wang, C.H.; Li, H.Y.; Shen, L.; Guo, W.B.; Ruan, S.P. Enhanced photovoltaic performance of tetrazine-based small molecules with conjugated side chains. *Acs Sustainable Chem. Eng.* **2017**, *5*, 8684-8692, doi:10.1021/acssuschemeng.7b01420.
